# COVID-19 Recognition Using Ensemble-CNNs in Two New Chest X-ray Databases

**DOI:** 10.3390/s21051742

**Published:** 2021-03-03

**Authors:** Edoardo Vantaggiato, Emanuela Paladini, Fares Bougourzi, Cosimo Distante, Abdenour Hadid, Abdelmalik Taleb-Ahmed

**Affiliations:** 1 Department of Innovation Engineering, University of Salento, 73100 Lecce, Italy; edoardo.vantaggiato@studenti.unisalento.it (E.V.); emanuela.paladini@studenti.unisalento.it (E.P.); 2 IEMN UMR CNRS 8520, Université Polytechnique Hauts de France, UPHF, 59300 Famars, France; faresbougourzi@gmail.com (F.B.); abdenour.hadid@ieee.org (A.H.); 3 Institute of Applied Sciences and Intelligent Systems, National Research Council of Italy, 73100 Lecce, Italy

**Keywords:** COVID-19, deep learning, convolutional neural network, Ensemble-CNNs, X-ray scans

## Abstract

The recognition of COVID-19 infection from X-ray images is an emerging field in the learning and computer vision community. Despite the great efforts that have been made in this field since the appearance of COVID-19 (2019), the field still suffers from two drawbacks. First, the number of available X-ray scans labeled as COVID-19-infected is relatively small. Second, all the works that have been carried out in the field are separate; there are no unified data, classes, and evaluation protocols. In this work, based on public and newly collected data, we propose two X-ray COVID-19 databases, which are three-class COVID-19 and five-class COVID-19 datasets. For both databases, we evaluate different deep learning architectures. Moreover, we propose an Ensemble-CNNs approach which outperforms the deep learning architectures and shows promising results in both databases. In other words, our proposed Ensemble-CNNs achieved a high performance in the recognition of COVID-19 infection, resulting in accuracies of 100% and 98.1% in the three-class and five-class scenarios, respectively. In addition, our approach achieved promising results in the overall recognition accuracy of 75.23% and 81.0% for the three-class and five-class scenarios, respectively. We make our databases of COVID-19 X-ray scans publicly available to encourage other researchers to use it as a benchmark for their studies and comparisons.

## 1. Introduction

Since the appearance of COVID-19 in the city of Wuhan, China, at the end of 2019, great efforts have been made to recognize this disease. Reverse Transcription Polymerase Chain Reaction (RT-PCR) is the definitive test for the recognition of COVID-19 disease. However, RT-PCR test is a time-consuming, laborious, and complicated manual process [1]. In addition, test kits are only available in limited numbers worldwide [1]. On the other hand, the rate of false negatives varies depending on how long the infection has been present. In [2], the false-negative rate was 20% when testing was performed five days after symptoms began, but much higher (up to 100%) earlier in the infection.

Chest X-ray scans show visual indexes associated with COVID-19 [3]. In addition, chest X-ray scans are a fast, effectivem and affordable test to identify COVID-19 infection [4]. Despite the availability of chest X-ray scans, an expert radiologist is needed to identify the COVID-19 infection. Because of the huge number of infections, the healthcare systems have already been overwhelmed around the world. Artificial Intelligence (AI) systems can provide an alternative solution for the automatic diagnosis of COVID-19 infections and differentiate them from other diseases [5].

Many Artificial Intelligence (AI) systems have proved their efficiency in medical images analysis, such as pneumonia detection [6] semantic segmentation [7]. Similarly, many AI systems based on deep learning have been proposed and their performance has shown promising results in the diagnosis of COVID-19 infection from chest X-ray images [4,5,8,9,10]. The ability of deep convolutional neural networks to extract relevant and high-level features directly from data makes them more powerful than Hand-crafted methods [11]. Hand-crafted methods are based on extracting the features using designed models [12].

Since the appearance of COVID-19, great efforts have been made to recognize COVID-19 infection from X-ray scans. However, this field has not achieved great progress in the recognition of COVID-19 infection as a real application, and this is due to two main drawbacks. The first drawback is the limitation of COVID-19 X-ray scans. The second drawback is that there are no unified protocols, classes, and data. In the literature, each work defines its own protocol, classes, and data, and this makes comparison between different methods difficult. In this work, we aim to unify the efforts in this field. First, we created a great number of COVID-19 X-ray scans. In addition, we defined two scenarios for differentiating COVID-19 scans from scans of other lung diseases in three-class and five-class scenarios. Furthermore, we defined train/val/test splits to allow better comparison between different methods. We make our databases of COVID-19 X-ray scans publicly available to encourage other researchers to use them as a benchmark for their studies. The main contributions of this paper are:

- We created the largest COVID-19 X-ray scan database, with 504 scans collected from open sources and 207 new scans collected from the Hospital of Tolga, Algeria.
- We proposed two scenarios to distinguish between COVID-19 disease, other lung diseases, and healthy cases. In the first scenario, we created a three-class X-ray scan database which consists of three classes: Normal, COVID-19, and other Pneumonia diseases. In the second scenario, we created five-class X-ray database which includes the following classes: Normal, COVID-19, Viral Pneumonia, Bacterial Pneumonia, and Lung Opacity No Pneumonia. Furthermore, we divided both databases into training, validation, and test sets. Most of the testing data classes were taken from new sources that were not used to create the training and validation sets.
- In order to distinguish between COVID-19 infection and other Lung diseases, we used deep learning architectures for both scenarios (three classes and five classes). In addition, we proposed an Ensemble-CNNs approach based on the trained deep learning architectures.
- We make our codes and databases of COVID-19 X-ray scans publicly available to encourage other researchers to use it as a benchmark for their studies. (https://github.com/Edo2610/Covid-19_X-ray_Two-proposed-Databases (accessed on 2 March 2021), https://www.kaggle.com/edoardovantaggiato/covid19-xray-two-proposed-databases (accessed on 27 February 2021)).

This paper is organized in following way: In Section 2, we describe some of the state-of-the-art works. Section 3 consists of our proposed evaluation scenarios, illustrations of our proposed databases, and a description of the used methods and evaluation metrics. Our proposed approach is presented in Section 4. Section 5 includes the experimental setup and the results of the two defined scenarios. We compare our results with the state-of-the-art methods in Section 6. Finally, concluding remarks are given in Section 7.

## 2. Related Works

Motivated by the success of deep learning methods in many computer vision tasks, most of the existing works for the recognition of COVID-19 infection from X-ray scans have used deep leaning methods [4,5,8,9,10,13].

Hemdan et al. [14] tested seven different CNN architectures, including VGG-19 [15], DenseNet-121 [16], Inception-V3 [17], ResNet-V2 [18], InceptionResNet-V2 [19], Xception [20], and Google MobileNet-V2 [21]. Their database contains only 25 COVID-19 cases and they consider a binary classification of positive and negative COVID-19 infection. Their results showed that the VGG-19 and DenseNet-121 models achieved the best performance, where both architectures reached a 0.91 F1-score for COVID-19 infection and 0.89 for non-COVID-19 infection.

Mangal et al. [22] used CheXNet [23], which was trained on the ChestX-ray8 database [24]. They used transfer learning to recognize the COVID-19 infection within three- and four-class scenarios. They achieved a promising result with a recognition rate for COVID-19 infection equal to 90.5% in the three-class scenario.

In [4], Yoo et al. proposed a deep learning-based decision-tree classifier based on three binary decisions. Each binary decision is a trained ResNet-18 [18] architecture:

- First decision tree classifies the input image as normal or abnormal. The accuracy of this decision tree is 98%.
- Second decision tree identifies abnormal images that contain signs of tuberculosis (TB) or not. The accuracy of this decision tree is 80%.
- Third decision tree classifies abnormal images that contain signs of COVID-19 or not. The accuracy of this decision tree is 95%.

M. Turkoglu proposed the COVIDetectioNet [1] framework, which consists of three steps. First, the pre-trained AlexNet architecture [25] is used with transfer learning. In second step, the trained Alexnet is used to extract deep features from all layers. These features are concatenated to produce the combined features. In the final step, the Relief algorithm [26] is used to select the most relevant features from the combined features, then they are fed to a Support Victor Machine (SVM) classifier [27]. His approach showed a promising result in the used database, which consists of three classes: COVID-19, Pneumonia, and Normal. In [13], I.D. Apostolopoulos et al. tested five CNN architectures, VGG-19 [15], MobileNet-v2 [21], Inception [17], Xception [20], and Inception-ResNet-v2 [19], on two databases which were collected from different public resources. From their obtained results, the VGG-19 and MobileNet architectures achieved the best performance compared with the other used CNN architectures. In [28], A. T. Sahlol proposed using deep features that were extracted from the Inception architecture and a swarm-based feature selection algorithm to recognize COVID-19 infection from the X-ray scans. Their approach achieved considerable improvement compared with the set of feature selection algorithms and CNNs architectures.

Table 1 summarises the mentioned state-of-the-art works, the used databases, and the obtained results. From this table, we can notice that the used databases are different from one work to another with a small number of X-ray scans, specially for the COVID-19 class. Moreover, each work defines different classes and evaluation protocols. This motivated us to collect the available COVID-19 X-ray scans, provide our own COVID-19 X-ray scans, and define the evaluation protocol and scenarios.

## 3. Methodology

In this section, we will discuss the proposed evaluation scenarios and databases. In addition, we will describe the used CNN architectures, loss functions, and evaluation metrics.

### 3.1. Evaluation Scenarios

Most of the literature studies have dealt with the recognition of two or three classes of COVID-19-related diseases using initially small databases [1,9,13,14,22]. In our work, two scenarios are investigated to distinguish COVID-19 infection from other Lung diseases. In the first scenario, we defined three classes, which are:

- Healthy.
- COVID-19.
- Other pneumonia diseases.

To train our models, we collected 504 X-ray scans for each class. In this scenario, we evaluated the performance of three most popular CNN architectures (Densnet-151, Inception-v3, and ResneXt-50) and our proposed Ensemble-CNNs approach. In the training phase, we divided the 504 X-ray scans of each class into training–validation splits (80%–20%). To train the deep learning models, we used data-augmentation techniques for the training split to gain 6048 augmented X-ray scans for each class. In the testing phase, we used 207 X-ray scans for each class, where the X-ray scans of COVID-19 were obtained from the Hospital of Tolga, Algeria. For the other image classes, we emphasized collecting them from new sources that were not used in the creation of the training and validation splits. Here, we aim to study the performance of the methods in different conditions, which can include variation in the illumination, contrast, and recording device used.

In the second scenario, we identified four classes of Lung Diseases and Normal. The classes of the second scenario are:

- Normal.
- COVID-19.
- Viral Pneumonia.
- Bacterial Pneumonia.
- Lung Opacity No Pneumonia.

Similar to the first scenario, we used 504 X-ray scans as training–validation splits (80%–20%), then the same data augmentation techniques were used for the training split. In the testing phase, we used 207 X-ray scans for each class, where the X-ray scans of COVID-19 were obtained from the Hospitals of Tolga, Algeria. Similar to the three-class database, we emphasized collecting the non-COVID-19 images from new sources that were not used for creating the training and validation splits. The goal is to study the performance of the methods in different conditions that can include variation in the illumination, contrast, and recording device used.

### 3.2. Databases

Most of the state-of-the-art databases for recognizing COVID-19 infection from X-ray scans consider just two or three classes. The two classes are COVID-19 and Healthy, which were used in [14]. Meanwhile, the three classes are COVID-19, Healthy, and Pneumonia, which were used in [1,9,22]. In our work, we investigated two scenarios.

In the first scenario, we considered three classes, which are COVID-19, Pneumonia, and Normal (or Healthy). Meanwhile, in the second scenario we considered five classes, where the Pneumonia can be classified into Bacterial Pneumonia and Viral Pneumonia. As a fifth class, we considered Lung Opacity Not Pneumonia disease, including all lung diseases that are not Pneumonia. To the best of our knowledge, this is the first time that five lung diseases including COVID-19 have been studied. We used the following resources to create our databases:

1. Ieee8023 COVID-19 Chest X-Ray database [29] is the main database used in most the state-of-the-art papers, from which we took 504 COVID-19 X-ray images. In this database, there are others classes but with few images. License: Apache 2.0, CC BY-NC-SA 4.0, CC BY 4.0
2. Chest X-Ray Images (Pneumonia) [30] from Kaggle that contains a lot of images for the classes Pneumonia and Normal. For Pneumonia images, there are two classes, which are Bacterial and Viral. License: CC BY 4.0
3. RSNA Pneumonia Detection Challenge [31] from Kaggle. From this source, we took only Normal and Pneumonia images. In the Pneumonia class there is no distinction between types. License: Open Source
4. CheXpert [32] is a large chest X-ray database from which we took Normal images, and it is the only database that includes Lung Opacity images. License: Public database
5. China CXR set and Montgomery set [33] are two public databases that contain both Normal as well as tuberculosis X-rays. We used tuberculosis images for the Bacterial Pneumonia class. License: Public database

In addition to the use of above open source databases, we collected 207 unpublished X-ray samples for the COVID-19 class from the Hospital of Tolga, Algeria. These COVID-19 scans were used as testing data. In addition, we selected 207 images as testing data for the other classes. Most of the testing data classes were taken from new sources that were not used to create the training data for both the three- and five-class scenarios.

#### 3.2.1. Three-Class COVID-19 Database

We created the three-class database using all the available COVID-19 scans. In order to create a balanced database, we selected 504 images for each class since there are just 504 COVID-19 samples that are publicly available. For training and validating our models, we randomly split the three-class database into training and validation splits (80%–20%).

Since Deep Learning methods require huge amounts of labeled data for training, which is actually not available for the COVID-19 class, we used data augmentation techniques to cope with this issue. By applying the following data augmentation techniques for the training split, we obtained 12 augmented images for each image:

- Color jitter with brightness = 0.2, contrast = 0.2.
- Padding of 10 applied on each border.
- Random Horizontal flip.
- Random Perspective with distortion scale = 0.5.
- Random Rotation from -30 to 30 degree.
- Random Crop making sure that the smaller size remains at least 224px.

Each of the first five data augmentation techniques has an application probability equal to 50%.

**Figure 1:**
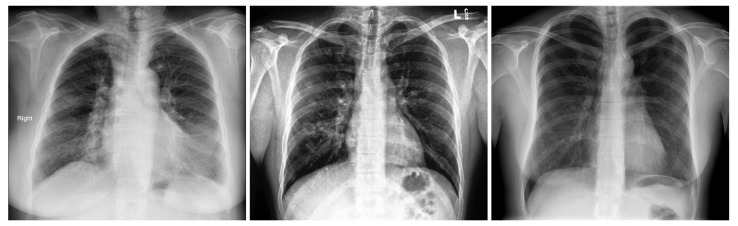
Samples from the three-class Covid-19 database: Covid-19 (**left**), Pneumonia (**center**), and Normal (**right**).

Table 2 summarizes the three-class database number of images by split and their resources. Figure 1 shows an X-ray example for each class of the three-class COVID-19 database.

#### 3.2.2. Five-Class Covid-19 Database

**Figure 2:**
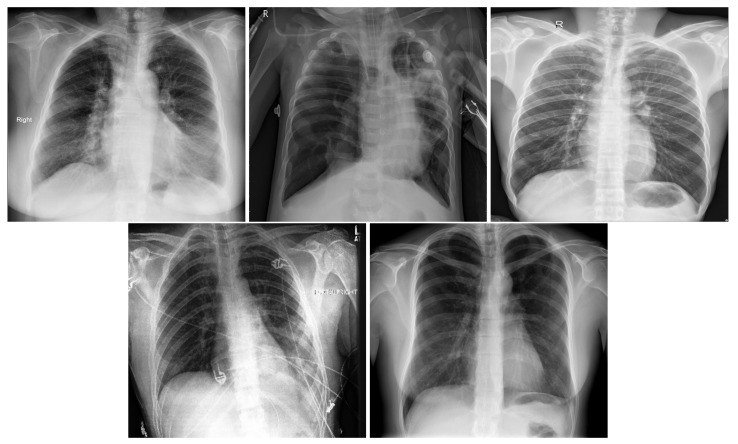
In order: COVID-19, Viral Pneumonia, Bacterial Pneumonia, Lung Opacity, Normal.

In order to distinguish between COVID-19 and the other lung diseases and healthy cases, we created a five-class COVID-19 database. In fact, COVID-19 is a viral pneumonia, so we aim to distinguish between Bacterial, Viral Pneumonia, COVID-19, and Healthy cases. In addition, we considered Lung Opacity Not Pneumonia diseases as the fifth class. Similar to the three-class COVID-19 database, we used data augmentation techniques to obtain augmented data to train our models. The same data augmentation techniques were applied for the training split to obtain 12 augmented images for each image. Table 3 summarizes the five-class COVID-19 database number of images by split and their resources. Figure 2 shows an X-ray example for each class of the five-class COVID-19 database.

### 3.3. CNN Architectures

In our experiments, we used three of the most powerful pre-trained CNN models, which are: ResNeXt-50 [34], Inception-v3 [17], and DenseNet-161 [16].

#### 3.3.1. ResNeXt-50

**Figure 3:**
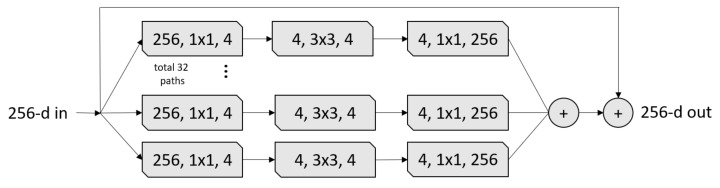
A ResneXt Module with cardinality = 32, with roughly the same complexity. A layer is shown as (# in channels, filter size, # out channels) [34].

The ResNeXt [34] architecture inherited its structure from three CNN architectures: VGG, ResNet, and Inception. From the VGG architecture, ResNext leveraged repeating layers to build a deep architecture model. ResNeXt uses the idea of shortcut from the previous layer to the next layer from the ResNet architecture. Similar to the Inception block, the ResNeXt block adopts a split-transform-merge strategy (branched paths within a single module), as shown in Figure 3. In the ResNeXt block shown in Figure 3, the input is split into a few lower-dimensional embeddings (by 1 × 1 convolutions) with 32 paths each for four channels, then all paths are transformed by the same topology filters of size 3×3. Finally, the paths are merged by summation. In our experiments, we used the ResneXt-50 pre-trained model, which was trained on ImageNet challenge database [25]. The ResNeXt-50 construction is summarized in Table 4.

#### 3.3.2. Inception-v3

Inception-v3 [17] is the third version of the Google Inception architecture family [35]. Since choosing the right kernel size is challenging for CNN architectures, Inception networks use filters with multiple sizes that operate on the same level, which makes the networks wider instead of deeper. In summary, Inception-v3 has several improvements over the previous versions, including:

1. Factorized convolutions;
2. Smaller convolutions;
3. Asymmetric convolutions;
4. Auxiliary classifier;
5. Grid size reduction.

In our experiments, we used the Inception-v3 pre-trained model, which was trained on the ImageNet challenge database [25]. The Inception-v3 architecture is summarized in Table 5.

#### 3.3.3. DenseNet-161

**Figure 7:**
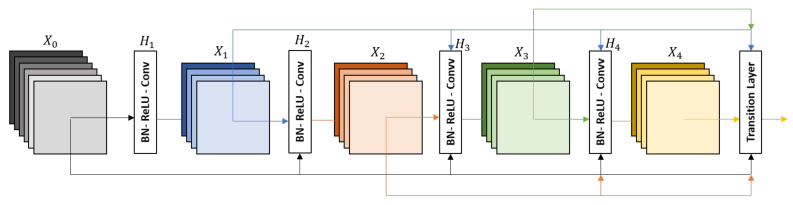
A 5-layer dense block with a growth rate of *k* = 4. Each layer takes all the preceding feature-maps as an input [16].

Densnet networks [16] seek to solve the problem of CNNs when going deeper. This is because the path for information from the input layer until the output layer (and for the gradient in the opposite direction) becomes so big that they can be lost before reaching the other side. G. Huang et al. [16] proposed to connect each layer to every other layers in a feed-forward fashion (as shown in Figure 7) to ensure maximum information flow between layers in the network. In our experiments, we used the DenseNet-161 pre-trained model, which was trained on the ImageNet challenge database [25]. The DenseNet-161 architecture is summarized in Table 6.

### 3.4. Loss Function

In our experiments, we used the Focal loss function [36], which was used for one-stage object detectors. Focal loss function has proven its efficiency in many classification tasks [37,38]. The multi-classes focal loss is formulated in the following equation:

(1) $$F_{fl}=-\sum_{i=1}^{C}{\left(1-y_{i}\right)}^{\gamma }t_{i}\;log\left(y_{i}\right),$$

where *C* denotes the number of categories, $t_{i}$ denotes a real probability distribution, $y_{i}$ denotes the probability distribution of the prediction, and γ is the focusing parameter which is used to control the rate at which easy examples are down-weighted.

In more detail, the Focal loss function applies a modulating term to the cross-entropy loss in order to focus learning on hard negative examples. It is a dynamically scaled cross entropy loss where the scaling factor decays to zero as confidence in the correct class increases. Intuitively, this scaling factor can automatically down-weight the contribution of easy examples during training and rapidly focus the model on hard examples.

### 3.5. Evaluation Metrics

To evaluate the performance of our models, we used six metrics: *Accuracy*, *Precision*, *Sensitivity*, *specificity*, *F1-score*, and *Area Under the ROC Curve (AUC)*. The *Accuracy* calculates the exact percentage of the correct predicted images with respect to the total images that were used. The formula for accuracy is as following:

(2) $$Accuracy=\frac{Number\;of\;correct\;prediction}{Total\;Number\;of\;predictions}\;\times 100.$$

The formulae of *Precision*, *Sensitivity*, *specificity*, and *F1-score* are defined by:

(3) $$Precision=\frac{TP}{TP+FP}\times 100,$$

(4) $$Sensitivity=\frac{TP}{TP+FN}\times 100,$$

(5) $$Specificity=\frac{TN}{TN+FP}\times 100,$$

(6) $$F1-\mathrm{score}=\frac{2\cdot Precision\cdot Sensitivity}{Precision+Sensitivity}\times 100.$$

The last evaluation metric is *Area Under the ROC Curve (AUC)*, which is calculated by adding successive trapezoid areas below the Receiver Operating Characteristic (ROC) curve. The ROC curve is created by plotting the true positive rate (TPR) against the false positive rate (FPR) at various threshold values. TPR and FPR are called also sensitivity/recall and 100 specificity, respectively.

## 4. Proposed Approach

**Figure 8:**
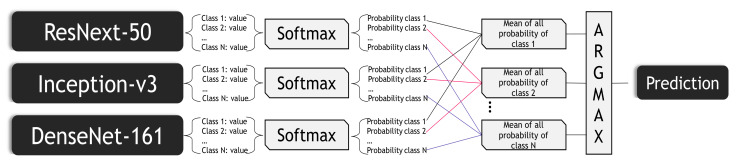
The architecture of our proposed Ensemble-CNNs approach.

For the three-class and five-class scenarios, we proposed an Ensemble-CNNs, which is based on the trained models, ResNeXt-50, Inception-v3, and DenseNet-161. Figure 8 illustrates our proposed approach. In our Ensemble-CNNs approach, the predicted class of each image is assigned using the average of the prediction probabilities of the three trained models. In more detail, the probabilities of the three models corresponding to all classes give the mean probability for all classes, then the argmax of the mean probabilities will assign the Ensemble-CNNs predicted class.

## 5. Experiments and Results

For three-class and five-class scenarios, we conducted the experiments using three powerful CNN architectures (ResNeXt-50, Inception-v3, and DenseNet-161) and our proposed Ensemble-CNNs approach. To evaluate the performance of these methods, we used the validation and testing splits. The main difference between both splits is that the validation split was created using the same sources as the training data, while the testing data were created from different sources. In this section, we will describe our experimental setup then the experiments of the three-class and five-class scenarios.

### 5.1. Experimental Setup

All the experiments were carried out on Pytorch [39] with a NVIDIA Device Geforce TITAN RTX 24 GB. All the networks were trained for 30 epochs with the Adam optimizer [40], the focal loss function with [36] γ=2, and batch size of 64. The initial learning rate was 1e-6 for 20 epochs, then leaning rate decreased to 1e-7 for the next 10 epochs. Active data augmentation was performed by normalizing, resizing, and cropping the input images in order to achieve the correct input size for each network; the input image size of the network was 299×299 pixels, meanwhile the DenseNet-161 and ReNeXt-50 input sizes were 224×224 pixels. For the normalization, the following values of mean and standard deviation were used for each channel of the image:

- mean: [0.485, 0.456, 0.406],
- std: [0.229, 0.224, 0.225].

Moreover, we added a dropout layer for both DenseNet-161 and ResNeXt-50 after the fully connected layer with a probability of 30%. Meanwhile, Inception-v3 already had a default dropout layer with a probability of 50%.

### 5.2. Three-Class Scenario Experiments

Table 7 and Table 8 summarise the results of the three-class scenario on the validation and testing data, respectively. From the results of the validation data, the best method is our proposed Ensemble-CNNs approach for all of the used evaluation metrics (*Accuracy*, *Precision*, *Sensitivity*, *specificity*, *F1-score*, and *AUC*). From Table 8, which contains the results of the testing data, ResneXt-50 achieved the best performance for the *Accuracy*, *Precision*, *Sensitivity*, *specificity*, and *F1-score* evaluation metrics, where it is slightly better than our proposed Ensemble-CNNs approach. Meanwhile, for the *AUC* evaluation metric DenseNet-161 achieved the best performance, and again DenseNet-161 was slightly better than our proposed Ensemble-CNNs approach. From these results, we notice that our proposed Ensemble-CNNs approach does not achieve the best result for all the evaluation metrics but still gives a better trade-off between different evaluation metrics’ results. In addition, we notice that the performance of the testing data is not good as that of the validation data. This is because the testing data sources are different from the training and validation ones, as shown in Table 2.

**Figure 9:**
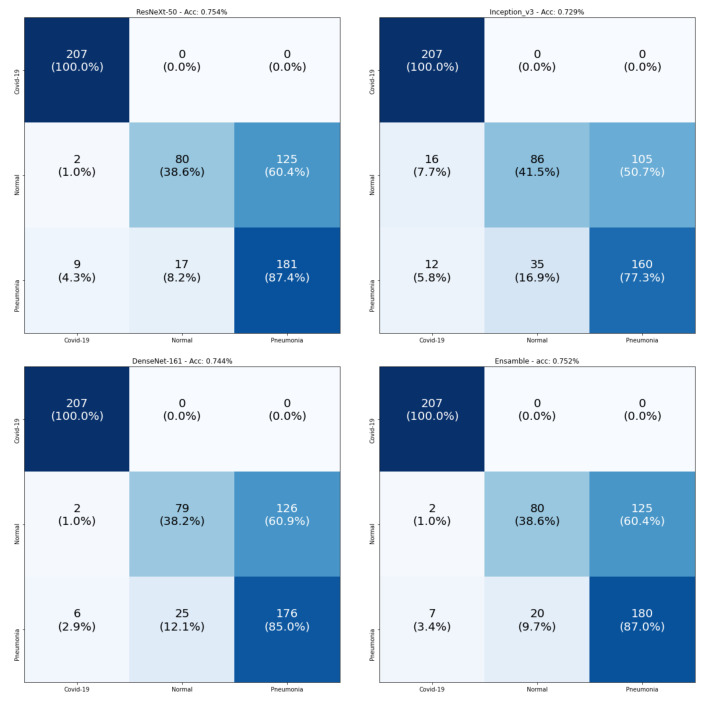
Confusion matrices of the three-class COVID-19 testing data using ResneXt-50, Inception-v3, DenseNet-161, and Ensemble-CNNs, respectively. The vertical axis is for the true classes and the horizontal axis is for the predicted classes.

Figure 9 consists of the confusion matrices of the testing data. The main observation is that all models achieved 100% for the classification of COVID-19 samples. The real confusion for all models was in distinguishing between the Normal and Pneumonia classes. Since all models achieved 100% in the recognition of COVID-19 samples, we checked the number of samples that were wrongly classified as COVID-19 for the testing split, as shown in Table 9. From this table, we observe that the best model was DenseNet-161, which had the smallest number of false positives, and our proposed Ensemble-CNNs approach was the second best one. From the above results, we conclude that our proposed approach is more stable in the classification of the three classes and the recognition of COVID-19.

### 5.3. Five-Class Scenario Experiments

Table 10 and Table 11 summarise the results of the five-class scenario with the validation and testing data, respectively. From these results, we notice that our proposed Ensemble-CNNs approach outperforms all of the three tested CNN architectures (ResNeXt-50, Inception-v3, and DenseNet-161) in both the validation and testing splits for all of the used evaluation metrics (*Accuracy*, *Precision*, *Sensitivity*, *specificity*, *F1-score*, and *AUC*). This proves the benefit of using the ensemble approach. As we noticed in three-class, the performance of the testing data was lower than that of the validation data. This is because the training and validation data were from the same sources for all classes. Meanwhile, most of the five-class testing data classes were from different sources, as shown in Table 3.

**Figure 10:**
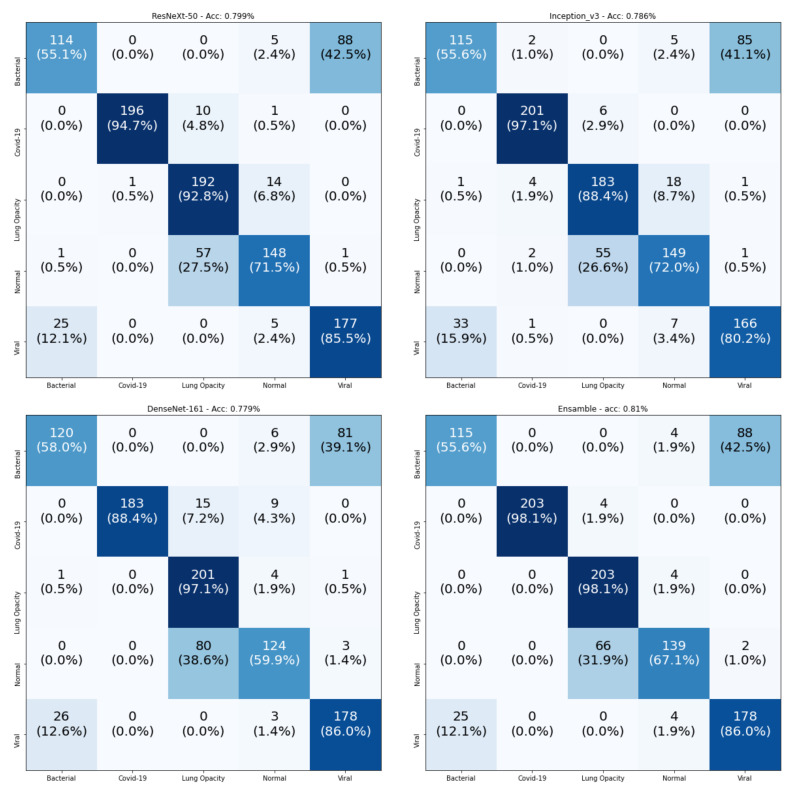
Confusion matrices of the five-class COVID-19 testing data using ResneXt-50, Inception-v3, DenseNet-161, and Ensemble-CNNs, respectively. The vertical axis is for the true classes and the horizontal axis is for the predicted classes.

To gain a better explanation for the recognition of the individual classes, Figure 10 contains the confusion matrices of the testing data. From these confusion matrices, we notice that the Ensemble-CNNs approach achieves the best performance in the recognition of COVID-19 samples (98.1%). In addition, the Lung Opacity No Pneumonia samples are well recognized by all models (the best one is Ensemble-CNNs, at 98.1%). This happened because all the samples for Lung Opacity No Pneumonia class were from a single source (we found only one source for this class). Table 12 shows a comparison between all four tested models in the recognition of the individual classes. From this table, we observe that our proposed approach is the best in the recognition of three classes out of five. This confirms the superiority of our approach compared with the other used CNN architectures.

### 5.4. Heatmap Representation

**Figure 11:**
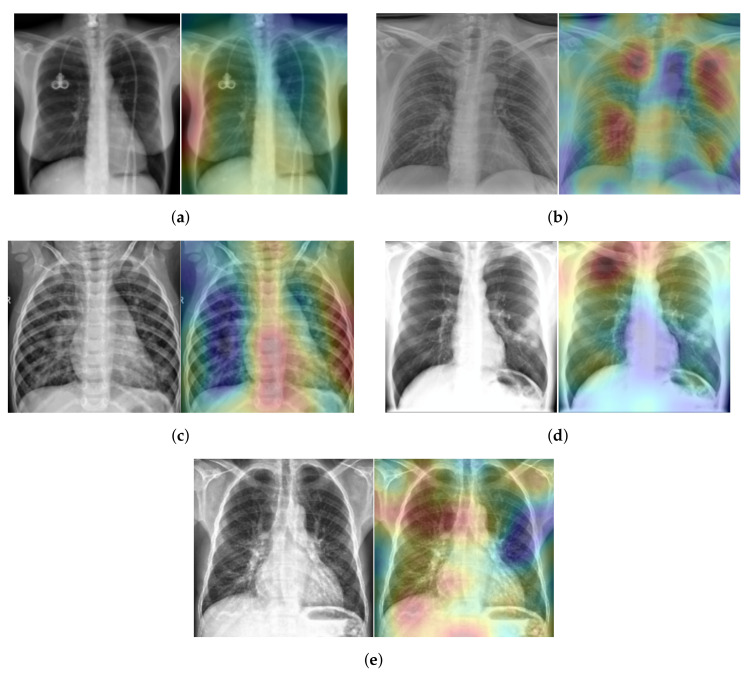
(**a**) Normal case, (**b**) COVID-19 case, (**c**) Viral Pneumonia case, (**d**) Bacterial Pneumonia case, (**e**) Lung Opacity case.

To explain our approach’s classification decision of different lung diseases from the X-ray scans, we used the *Randomized Input Sampling for Explanations* (RISE) approach [41]. Figure 11 shows the heatmap of five X-ray scans, where each scan has a different class. These X-ray scans were taken from the testing split of our five-class COVID-19 database. In Figure 11, the red color indicates the greater importance of the corresponding region to our model and the blue color indicates a lower importance. For the Healthy case (Figure 11a), most of the X-ray scan regions have a blue color, which indicates that all regions have the same importance as our approach, since there is no infection. Meanwhile, for the COVID-19, Viral Pneumonia, Bacterial Pneumonia, and Lung Opacity cases (Figure 11b–e), our approach gave more attention to the lung regions (red color), which correspond to the real regions where the infection occurs.

## 6. Discussion

Since the state-of-the-art works have no unified data, classes, or evaluation protocols, it is hard to compare different methods. In Table 13, we tried to compare the recognition of COVID-19 in our approach and that of some state-of-the-art methods. From this table, we notice that our approach has achieved a high performance in the recognition of COVID-19 in both scenarios (three and five classes), despite the fact that we used a new source of scans for the testing: Algeria. From other hand, the distinguishing between other lung diseases and normal cases is still challenging and need more improvement. It should be mentioned that the number of X-ray scans used for the training CNN architecture is very limited (404 X-ray scans for each class). One possible solution to improve the performance is to use more X-ray scans for each class.

Since we evaluated three CNN architectures and our proposed Ensemble-CNNs approach on our proposed new databases and scenarios, it is unfair to compare the complexity of our approach with the state-of-the-art methods. Table 14 contains the required time to test a single X-ray scan for the evaluated three CNN architectures and our approach for the three-class and five-class scenarios. From Table 14, we notice that the required time is very trivial for all the evaluated methods. This proves the efficiency of using X-ray scans for the recognition of COVID-19 infection compared with currently used tests, such as RT-PCR.

## 7. Conclusions

In this paper, we created two databases to distinguish between COVID-19 infection and other lung diseases from X-ray scans. In the first database, we considered three classes, which are Healthy, COVID-19, and Pneumonia. In the second database, we considered five classes, which are Healthy, COVID-19, Viral Pneumonia, Bacterial Pneumonia, and Lung Opacity No Pneumonia. In both databases, we collected public databases and used them as training and validation splits. However, we used new COVID-19 scans as testing images. Moreover, the testing splits of the other classes were collected from different sources.

To distinguish between different lung diseases in both scenarios, we evaluated three CNN architectures (ResNeXt-50, Inception-v3, and DenseNet-161) and proposed an Ensemble-CNNs approach. Since the CNN architectures require huge amounts of labelled data for training, we used data augmentation to cope with this limitation. The obtained results showed that our approach outperformed the CNN architectures. Our proposed Ensemble-CNNs achieved a high performance in the recognition of COVID-19 infection, resulting in accuracies of 100% and 98.1% in three-class and five-class scenarios, respectively. In addition, our approach achieved promising results in the overall recognition accuracy—75.23% and 81.0% for the three-class and five-class scenarios, respectively.

As future work, we are working on collecting more COVID-19 X-ray scans from hospitals. Moreover, we are planning to define more lung disease classes depending on the available X-ray scans. On the other hand, we are planing to use more powerful CNN architectures in our Ensemble approach. In addition, combining deep features of different architectures is an interesting approach that can improve the performance.

## Figures and Tables

**Figure 4:**
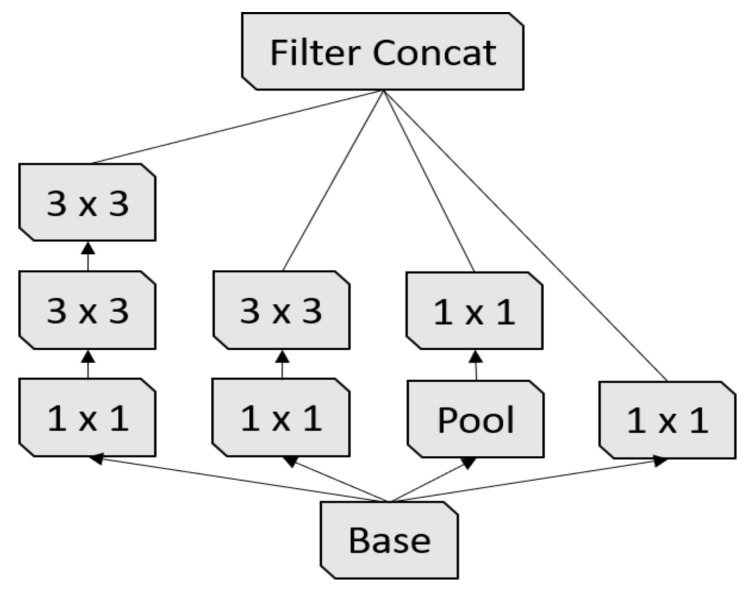
First Inception module [17].

**Figure 5:**
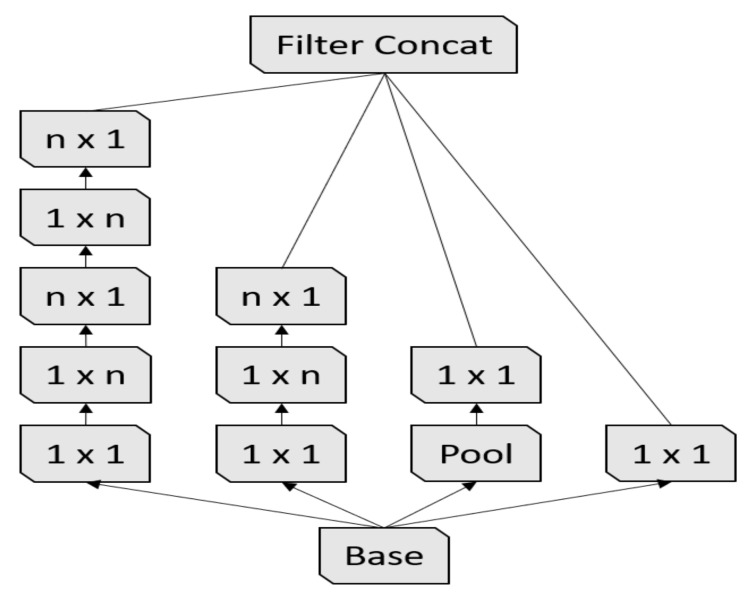
Second Inception module [17].

**Figure 6:**
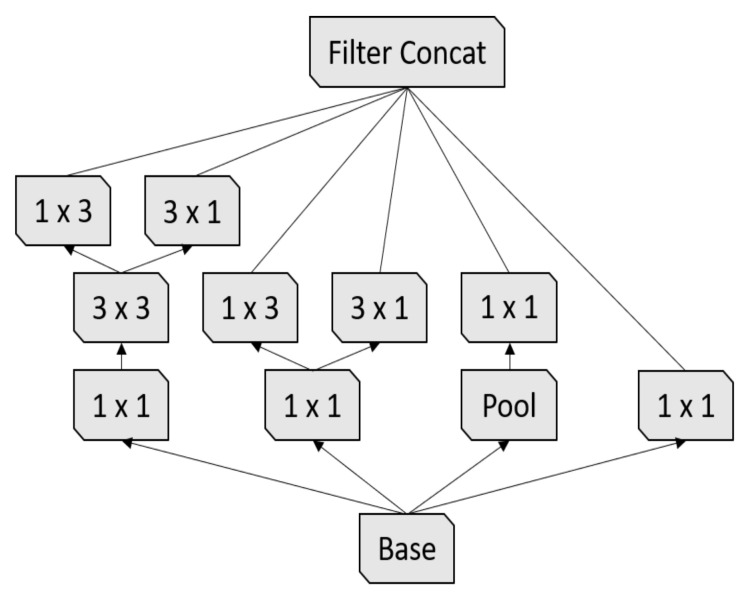
Third Inception module [17].

**Table 1:** State-of-the-art works summary.

Reference	Database	Splitting	Model	F1-Score
Hemdan et al. [14], 2020	- 25 Covid-19 - 25 Normal	- 40% Train - 40% Val - 20% Test	VGG-19 ResNet-v2 DenseNet-201	90 67 90
Apostolopoulos et al. [13], 2020	- 224 Covid-19 - 714 Pneumonia (400 bacterial + 314 viral) - 504 Normal	10-folds cross-validation	VGG-19 MobileNet-v2 Inception Xception Inception-ResNet v2	93.48 92.85 92.85 92.85 92.85
Karim et al. [9], 2020	- 259 Covid-19 - 8614 Pneumonia - 8066 Normal	5-folds cross-validation	VGG-16 VGG-19 ResNet-18 ResNet-34 DenseNet-161 DenseNet-201	76.1 92.5 92.1 86.1 94.5 90.5
Mangal et al. [22], 2020	- 155 Covid-19 - 4273 Pneumonia - 1583 Normal	- 88.5% Train - 0.6% Val - 10.9% test	CovidAID	92.3
Yoo et al. [28], 2020	- 585 Normal - 442 Abnormal - 492 TB - 492 Non TB - 120 Covid-19	- 83.3% Train - 16.7% Test	AXIR 1 AXIR 2 AXIR 3 AXIR 4	98 80 100 89
Turkoglu [1], 2020	- 219 Covid-19 - 4290 Pneumonia - 1583 Normal	10-folds cross-validation	COVIDetectioNet	99.18
A. T. Sahlol et al. [28], 2020	- 200 Covid-19 - 1675 Non-Covid	Unknown	FO-MPA	99.6
A. T. Sahlol et al. [28], 2020	- 219 Covid-19 - 1341 Non-Covid	Unknown	FO-MPA	99

**Table 2:** Three-class COVID-19 database sources and statistics.

Class	Train Set Original + Augmented	Validation Set	Test Set
Covid-19	404 [29] + 4848	100 [29]	207 **[Our]**
Pneumonia	404 [30] + 4848	100 [30]	207 [32]
Normal	404 [30,31] + 4848	100 [30,31]	207 [32]
Total	1212 + 14,544	300	621

**Table 3:** Five-Class COVID-19 database sources and statistics.

Class	Train Set Original + Augmented	Validation Set	Test Set
Covid-19	404 [29] + 4848	100 [29]	207 **[Our]**
Bacterial Pneumonia	404 [30,33] + 4848	100 [30,33]	207 [29]
Viral Pneumonia	404 [29,30] + 4848	100 [29,30]	207 [30]
Lung Opacity not Pneumonia	404 [32] + 4848	100 [32]	207 [32]
Normal	404 [30,31,32] + 4848	100 [30,31,32]	207 [33]
Total	2020 + 24,240	500	1035

**Table 4:** ResneXt-50 with a 32 × 4*d* template. Inside the brackets is the shape of a residual block, and outside the brackets is the number of stacked blocks on a stage. (C = 32) suggests grouped convolutions with 32 groups [34].

Stage	Output	ResneXt-50 (32×4d)
conv1	112×112	7×7×, 64, stride 2
conv2	56×56	3×3 max pool, stride 2
		$\left[\begin{array}{c} 1\times 1,128\\ 3\times 3,128,C=32\\ 1\times 1,256 \end{array}\right]$ × 3
conv3	28×28	$\left[\begin{array}{c} 1\times 1,256\\ 3\times 3,256,C=32\\ 1\times 1,512 \end{array}\right]$ × 4
conv4	14×14	$\left[\begin{array}{c} 1\times 1,512\\ 3\times 3,512,C=32\\ 1\times 1,1024 \end{array}\right]$ × 6
conv5	7×7	$\left[\begin{array}{c} 1\times 1,1024\\ 3\times 3,1024,C=32\\ 1\times 1,2048 \end{array}\right]$ × 3
	1×1	global average pool 1000-d fc, softmax

**Table 5:** Inception-v3 architecture [17].

Type	Parch Size/Stride or Remark	Input Size
conv	3×3/2	299×299×3
conv	3×3/1	149×149×32
conv padded	3×3/1	147×147×32
pool	3×3/2	147×147×64
conv	3×3/1	73×73×64
conv	3×3/2	71×71×80
conv	3×3/1	35×35×192
3× Inception M1	As in Figure 4	35×35×288
5× Inception M2	As in Figure 5	17×17×768
2× Inception M3	As in Figure 6	8×8×1280
pool	8×8	8×8×2048
linear	logits	1×1×2048
softmax	classifier	1×1×1000

**Table 6:** DenseNet-161 architecture, the growth rate is k=48. Note that each “conv” layer shown in the table corresponds with the sequence BN-ReLU-Conv [16].

Layers	Outsize	DenseNet-161 (k=48)
Convolution	112×112	7×7× conv, stride 2
Polling	56×56	7×7× max pool, stride 2
Dense Block 1	56×56	$\left[\begin{array}{c} 1\times 1\mathrm{conv}\\ 3\times 3\mathrm{conv} \end{array}\right]$ × 6
Transition	56×56	1×1 conv
Layer 1	28×28	2×2 average pool, stride 2
Dense Block 2	28×28	$\left[\begin{array}{c} 1\times 1\mathrm{conv}\\ 3\times 3\mathrm{conv} \end{array}\right]$ × 12
Transition	28×28	1×1 conv
Layer 2	14×14	2×2 average pool, stride 2
Dense Block 3	14×14	$\left[\begin{array}{c} 1\times 1\mathrm{conv}\\ 3\times 3\mathrm{conv} \end{array}\right]$ × 36
Transition	14×14	1×1 conv
Layer 1	7×7	2×2 average pool, stride 2
Dense Block 4	7×7	$\left[\begin{array}{c} 1\times 1\mathrm{conv}\\ 3\times 3\mathrm{conv} \end{array}\right]$ × 24
Classification	1×1	7×7 global average pool
Layer		1000D fully connected, softmax

**Table 7:** The experimental results for the validation data of our proposed three-class COVID-19 database.

Model	Accuracy (%)	Precision (%)	Sensitivity (%)	Specificity (%)	F1-Score (%)	AUC
ResNeXt-50	95.67 ± 2.30	95.05	94.99	97.50	95.00	0.9625
Inception-v3	92.67 ± 2.95	93.38	93.38	93.00	93.01	0.9475
DenseNet-161	95.00 ± 2.47	95.33	95.33	94.99	94.99	0.9625
Ensemble-CNNs	**96.67 ± 2.03**	**96.77**	**96.67**	**98.33**	**96.66**	**0.9750**

**Table 8:** The experimental results for the testing data of our proposed three-class COVID-19 database.

Model	Accuracy (%)	Precision (%)	Sensitivity (%)	Specificity (%)	F1-Score (%)	AUC
ResNeXt-50	**75.42 ± 3.39**	**78.85**	**75.36**	**87.68**	**73.53**	0.8080
Inception-v3	72.92 ± 3.50	73.18	72.95	71.30	72.92	0.7971
DenseNet-161	74.44 ± 3.43	76.84	74.40	87.20	72.69	0.8152
Ensemble-CNNs	75.23 ± 3.40	78.28	75.20	87.60	73.43	**0.8140**

**Table 9:** False positive of the testing three-class split for the COVID-19 class.

Model	False Positive		Total
	Normal	Pneumonia	
ResNeXt-50	2	9	11
Inception_v3	16	12	28
DenseNet-161	2	6	8
Ensemble-CNNs	2	7	9

**Table 10:** The experimental results of the validation data of our proposed five-class COVID-19 database.

Model	Accuracy (%)	Precision (%)	Sensitivity (%)	Specificity (%)	F1-Score (%)	AUC
ResNeXt-50	81.24 ± 3.42	83.96	83.60	86.40	83.63	0.8870
Inception-v3	80.55 ± 3.47	82.57	83.20	85.80	83.07	0.8950
DenseNet-161	82.20 ± 3.35	84.50	84.40	89.85	84.40	0.8963
Ensemble-CNNs	**93.2 ± 2.21**	**93.93**	**93.20**	**98.30**	**93.25**	**0.9575**

**Table 11:** The experimental results of the testing data of our proposed five-class COVID-19 database.

Model	Accuracy (%)	Precision (%)	Sensitivity (%)	Specificity (%)	F1-Score (%)	AUC
ResNeXt-50	79.94 ± 2.44	81.42	79.90	84.79	79.57	0.8617
Inception-v3	78.62 ± 2.50	79.35	78.65	84.66	78.31	0.8665
DenseNet-161	77.93 ± 2.53	80.43	77.51	84.47	77.93	0.8744
Ensemble-CNNs	**81.00 ± 2.39**	**82.99**	**82.96**	**85.24**	**81.49**	**0.8810**

**Table 12:** The best classification method for each class of the testing split of the five-class scenario.

Class	Model	Best Accuracy (%)
Bacterial Pneumonia	DenseNet-161	58.0
COVID-19	Ensemble-CNNs	98.1
Lung Opacity Not Penumonia	Ensemble-CNNs	98.1
Normal	Inception-v3	72.0
Viral Pneumonia	DenseNet-161 & Ensemble-CNNs	86.0

**Table 13:** Comparison between our results and the state-of-the-art results for the recognition of COVID-19 infection.

Reference	Classes	Total COVID-19 Samples	Test COVID-19 Samples	COVID-19 Accuracy
Hemdan et al. [14]	2	25	5	100%
Apostolopoulos et al. [13]	3	224	24	98.66%
Karim et al. [9]	3	259	137	90.5%
Mangal et al. [22]	3	155	30	100%
Yoo et al. [4]	4	120	42	95%
Turkoglu et al. [1]	3	219	20	100%
Ensemble-CNNs (our)	3	711	207	100%
Ensemble-CNNs (our)	5	711	207	98.1%

**Table 14:** Testing time for the evaluated CNN architecture and our proposed Ensemble-CNNs approach for three-class and five-class scenarios.

Model	Scenario	
	Three-Class Scenario (s)	Five-Class Scenario (s)
ResNeXt-50	0.024985	0.029988
Inception-v3	0.014991	0.065963
DenseNet-161	0.037979	0.038977
Ensemble-CNNs	0.077955	0.134928

## Data Availability

The used datasets were obtained from publically open source datastes from: 1 ieee8023/covid-chestxray-dataset https://github.com/ieee8023/covid-chestxray-dataset (accessed on 2 March 2021); 2 Chest X-Ray Images (Pneumonia) from Kaggle https://www.kaggle.com/paultimothymooney/chest-xray-pneumonia (accessed on 2 March 2021); 3 RSNA Pneumonia Detection Challenge from Kaggle https://www.kaggle.com/c/rsna-pneumonia-detection-challenge (accessed on 2 March 2021); 4 A Large Chest X-Ray Dataset - CheXpert https://stanfordmlgroup.github.io/competitions/chexpert/ (accessed on 2 March 2021); 5 NLM-MontgomerySet https://lhncbc.nlm.nih.gov/LHC-publications/pubs/TuberculosisChestXrayImageDataSets.html (accessed on 2 March 2021); 6 NLM-ChinaCXRSet https://lhncbc.nlm.nih.gov/LHC-publications/pubs/TuberculosisChestXrayImageDataSets.html (accessed on 2 March 2021); 7 Algeria Hospital of Tolga https://github.com/Edo2610/Covid-19_X-ray_Two-proposed-Databases/tree/main/Datasets/5-classes/Test/Covid-19 (accessed on 2 March 2021).

## Abbreviations

The following abbreviations are used in this manuscript:

CNN	Convolutional Neural Network
RT-PCR	Reverse Transcription Polymerase Chain Reaction
SVM	Support Vector Machine
TP	True Positive
FP	False Positive
FN	False Negative
AUC	Area Under the ROC Curve
ROC	Receiver Operating Characteristic
